# Risk stratification in transvenous lead extraction: Current models and clinical applications

**DOI:** 10.1016/j.hroo.2025.10.016

**Published:** 2025-11-04

**Authors:** Alphonsus C. Liew, Vishal Mehta, Nadeev Wijesuriya, Sandra Howell, Felicity de Vere, Steven Niederer, Christopher Aldo Rinaldi

**Affiliations:** 1 School of Biomedical Engineering and Imaging Sciences, King’s College London, London, United Kingdom; 2 Department of Cardiology, Guy’s and St Thomas’ NHS Foundation Trust, London, United Kingdom; 3 National Heart and Lung Institute, Imperial College London, London, United Kingdom; 4 The Alan Turing Institute, London, United Kingdom; 5 Heart Vascular and Thoracic Institute, Cleveland Clinic, London, United Kingdom

**Keywords:** Lead extraction, Transvenous, Risk stratification, Pacemaker, Implantable cardiac defibrillator, Complication, Mortality

## Abstract

Transvenous lead extractions have been increasingly performed in the last decade, in line with the rise in cardiac implantable electronic device implantation. Contemporary transvenous lead extraction registries have demonstrated high procedural success rates and low major complication and mortality rates. However, procedural risk remains highly variable and is dependent on several factors. This highlights the importance of risk stratification to facilitate adequate procedural planning including procedural setting (operating room, hybrid laboratory, or device laboratory), presence of surgical backup, and tools (simple mechanical vs powered sheaths) required. Furthermore, risk stratification allows for upfront decisions to be made regarding lead management (extraction or abandonment) and frank discussions to be had with patients regarding the risk of serious complications and the probability of procedural success. To date, several risk prediction tools have been mooted to predict procedural and/or postprocedural risk, as well as procedural difficulty and need for advanced techniques or tools. In this review, we will summarize and compare the different risk prediction tools available. We will provide practical recommendations on the use of risk prediction tools to improve procedural preparation and minimize mortality from procedural complications.

## Graphical abstract

**Figure:**
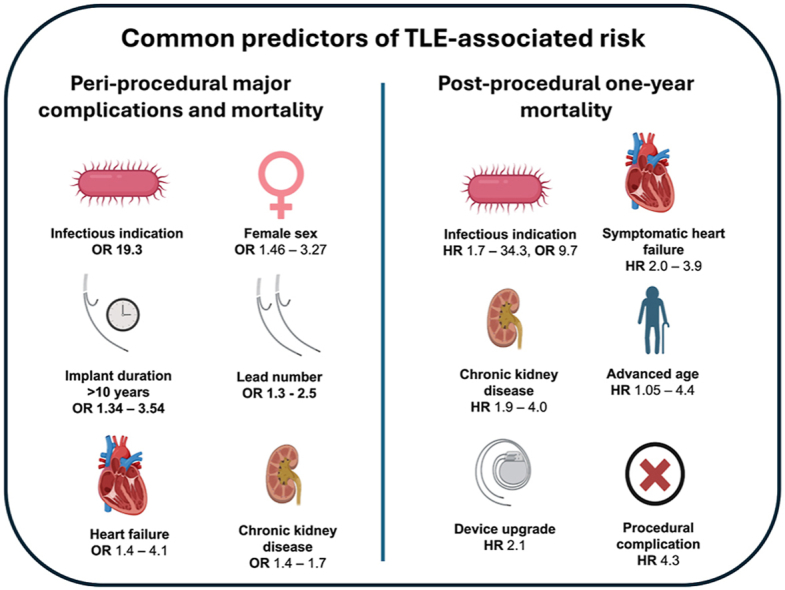

**Key Findings**

▪ Although overall transvenous lead extraction (TLE) procedural major complication and mortality rates are low, individual risk is highly variable.
▪ Postprocedural mortality remains substantial despite high acute procedural success rates and noninfectious indication.
▪ Risk stratification tools provide a framework to improve resource allocation, reduce surgical response time, inform discussions with patients, and guide the intensity and duration of follow-up after TLE.
▪ The ELECTRa registry outcome score risk model takes into account lead and nonlead parameters, has been externally validated using the large ELECTRa registry, and can effectively predict procedural mortality, major complications, and use of powered sheaths.
▪ Patients with risk factors for higher post-TLE mortality, such as infectious indication, renal impairment, advanced age, symptomatic heart failure, device upgrade indication, and acute procedural complication, should be monitored closely with repeat clinical assessments and blood tests.

## Introduction

The number of transvenous lead extraction (TLE) procedures has increased rapidly over the last decade.^1^^,^^2^ Contemporary TLE registries have demonstrated mortality to be as low as 0.5%–0.9% and major complication rates as low as 1.7%–2.3%.^1^^,^^2^ However, complication rates should be interpreted and communicated to potential extraction candidates with caution given that the risk of lead extraction is highly individualized. For example, patients with infectious indication have a much higher risk of major complications and mortality, with a reported 1-year mortality between 7% and 20%.^3^, ^4^, ^5^, ^6^, ^7^This highlights the importance of risk stratification prior to undertaking TLE to inform procedural preparation, optimize resource allocation, ensure timely provision of surgical backup, and guide intensity postprocedural care and monitoring.

To date, a multitude of risk stratification models have been proposed. In this review, we start with a case vignette in which procedural planning was guided by risk stratification. We systematically explore various risk stratification tools and discuss their utility in communicating risk to patients and informing postprocedural monitoring. We offer practical recommendations on procedural preparation, including the location of TLE, and postprocedural monitoring based on stratified risk. The 2018 European Heart Rhythm Association (EHRA) and 2017 Heart Rhythm Society (HRS) expert consensus on lead extraction definition of major complications, procedural success, and clinical success were used in writing this review.^8^^,^^9^

## Case vignette

A 68-year-old man who had a dual-chamber pacemaker implanted 20 years ago for second-degree atrioventricular block and a subsequent generator change 6 years ago presented to his local hospital 3 years ago with erythema around his pacemaker site. The impression was that the erythema was caused by pressure exerted by the underlying leads and the patient underwent an uncomplicated wound revision to reposition the subcutaneous leads. 2 years later, the patient presented with a similar issue. A computed tomography (CT)–positron emission tomography scan performed at the time demonstrated low-grade uptake around the pacemaker generator, which was suspicious for pocket infection. Shortly after, the patient developed pocket erosion, and a wound swab was positive for *Pseudomonas aeruginosa*. His case was referred to our institution and discussed at our lead extraction multidisciplinary team meeting. The patient was risk stratified using the ELECTRa registry outcome score (EROS) risk stratification score and was deemed to be high risk (EROS 3) of procedural risk, owing to the age of his pacemaker leads (>15 years). Accordingly, the procedure was performed in a hybrid theater with surgical backup.

A superior approach was adopted with preemptive placement of a stiff wire in the superior vena cava (SVC) and vascular occlusion balloon in the inferior vena cava. Lead locking devices were used in the first instance but were unable to free the leads. After this, a laser sheath was used on the right ventricular lead up to the SVC junction before the locking stylet broke. A femoral approach was subsequently attempted with needle eye snaring and lead extender with continuous use of a laser sheath. This finally resulted in the complete extraction of all lead material. The patient subsequently underwent successful implantation of a leadless pacemaker in the same sitting. Postprocedural echocardiography revealed no pericardial effusion or tricuspid valve damage. Postprocedural chest radiograph showed complete removal of all lead material and good positioning of the leadless pacemaker. Follow-up at 6 weeks revealed a well-healed wound and satisfactory parameters of the leadless pacemaker.

This case demonstrates how a risk stratification model effectively predicted the need for advanced extraction tools, including a laser sheath and femoral snaring. It also informed the prophylactic placement of an endovascular occlusion balloon and guided both the procedural setting for TLE and the availability of surgical backup. Although no major complications necessitating emergent thoracotomy occurred, the prolonged lead dwell time indicated the likelihood of dense adhesions and an elevated risk of SVC injury. In such a scenario, the preemptive placement of a vascular occlusion balloon—prompted by the high-risk score—could have substantially reduced the risk of catastrophic bleeding and procedural mortality.

## Procedural risk stratification models

To date, several procedural risk stratification models have been proposed to assess procedural risk in TLE. Below, we provide an overview of each model, in chronological order.

### Fu et al**^10^** (2015)

Fu et al^10^ conducted a retrospective study on 652 patients who underwent TLE.^10^ Major complication rates were reported at 1.9%. Logistic regression analysis identified lead duration to be a significant predictor of major complications (odds ratio [OR] per year 1.22; 95% confidence interval [CI] 1.13–1.33; *P* < .001). Subsequently, a risk stratification model was proposed based on lead dwell times and their predicted risk of rescue surgery (Table 1). Based on this, Fu et al^10^ modified their practice to perform low- and moderate-risk TLE procedures in a well-equipped electrophysiology (EP) laboratory with surgical backup and a wire in the SVC for moderate-risk procedures to allow for prompt deployment of an endovascular balloon in the event of SVC laceration. Procedures estimated to be high risk were performed in the operating room with a standby surgeon available to undertake emergent thoracotomy or sternotomy if required.

**Table 1:** The associated risks of the rescue strategy with different risk groups and the suggested location for performing the procedure

Level of risk	Criteria	Risk of rescue surgery	Suggested location of TLE
Low risk	- Oldest lead <1 y	0%	EP lab
Medium risk	- Oldest pacemaker lead ≤10 y or - Oldest ICD lead ≤5 y	1.2%	EP lab with preemptive wire in SVC for endovascular balloon deployment when required
High risk	- Oldest pacing lead >10 y or - Oldest ICD lead >5 y	5.3%	Operating room with a cardiac surgeon present on standby

EP lab = electrophysiology laboratory; ICD = implantable cardioverter-defibrillator; SVC = superior vena cava; TLE = transvenous lead extraction.

### Kancharla et al**^11^** (2019)

Based on risk factors identified from a retrospective review of patients who underwent TLE at the Mayo Clinic (Rochester, Minnesota), Kancharla et al^11^ devised a risk stratification model to prospectively allocate 187 patients undergoing TLE into intermediate- and high-risk groups (Table 2).^11^ The intermediate-risk group had their extraction procedure performed in the EP laboratory with surgical backup, and the high-risk group had their procedure performed in the hybrid laboratory. The surgical backup for the intermediate-risk group was defined as a dedicated surgeon who would be immediately available to perform an emergency surgical procedure in the event of SVC or myocardial tear.

**Table 2:** Criteria for intermediate- and high-risk groups based on Kancharla et al^11^

Risk group	Risk factors for TLE
Intermediate risk	Pacemaker lead age ≤10 y
	ICD lead age ≤5 y
High risk	Pacemaker lead age >10 y. ICD lead age >5 y or Pacemaker lead age ≤10 y, ICD lead age ≤5 y with any of the following: - Congenital heart disease - Initial implant when the patient was <15 y old - Hemodialysis - Chest radiograph or CT scan showing calcified SVC or myocardium adjacent to the lead - Active sepsis - Heart failure with NYHA class IV

CT = computed tomography; ICD = implantable cardioverter-defibrillator; NYHA = New York Heart Association; SVC = superior vena cava; TLE = transvenous lead extraction.

Major complications occurred in 6.9% in the high-risk group vs 0% in the intermediate-risk group (*P* = .007). The high-risk group demonstrated a trend toward more procedural deaths (2.7%) and in-hospital deaths (8.3%) than the intermediate-risk group (0% and 2.6% respectively). There was no significant difference in 1-year mortality between the groups.

### Risk stratification prior to lead extraction protocol (2019)^12^

**Figure 1:**
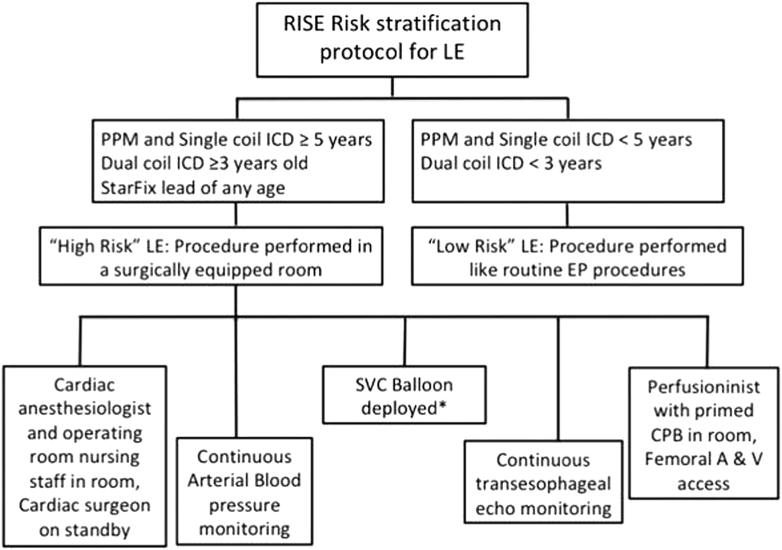
RISE protocol Afzal et al.^12^ Reproduced with permission from John Wiley and Sons. A = artery; CBP = cardiopulmonary bypass; EP = electrophysiology; ICD = implantable cardioverter-defibrillator; LE = lead extraction; PPM = permanent pacemaker; RISE = risk stratification before lead extraction; SVC = superior vena cava; V = vein.

Afzal et al^12^ retrospectively analyzed 449 patients who underwent TLE at their institution to identify risk factors of major adverse cardiac events, defined as emergent open-heart surgery, pericardial effusion requiring draining, hemothorax requiring draining, stroke, and death. The identified risk factors were used to prospectively dichotomize 751 patients into low- and high-risk groups for TLE based on lead dwell time, presence of dual-coil implantable cardioverter-defibrillator (ICD), and presence of StarFix lead of any age. The high-risk group underwent their TLE procedure in the operating room or hybrid laboratory with a cardiac surgeon on the premises, whereas the low-risk group underwent their extraction procedure in the EP laboratory as a routine procedure. The risk stratification before lead extraction (RISE) protocol for both risk groups is presented in Figure 1.

The authors demonstrated a 2-fold reduction in major adverse cardiac events in the post-RISE protocol group compared with the pre-RISE protocol group (1.6% vs 3.34%, respectively; *P* = .04). Similarly, there was a reduction in death after implementation of the RISE protocol (post-RISE 0.13% vs pre-RISE 0.89%; *P* = .04). The time taken to initiate reparative surgery in the event of a complication was also significantly shorter in the post-RISE group than the pre-RISE group (16 minutes vs 38 minutes; *P* = .02).

### SAFeTY score (2020)^13^

Jacheć et al^13^ retrospectively analyzed 2049 patients undergoing TLE, without the use of powered sheaths, at a single center to identify the predictors of major procedural complications. A risk stratification model was proposed to predict the risk of major procedural complications based on variables including total sum of lead dwell times of >16.5 years (6.095 points), patient’s age of <30 years at initial implant (2.174 points), hemoglobin concentration of <11.5 g/dL (2.291 points), female sex (2.74 points), and number of previous cardiac implantable electronic devices procedures (1.364 points per procedure). The risk of major complications was then predicted using the following formula:

$$\text{Risk}\;\text{of}\;\text{major}\;\text{complications}\;\left(\%\right)=\frac{100}{\left(1+\frac{6.44}{1.3213^{x}}\right)}$$

where “x” is the sum of points obtained.

Based on the predicted risk of major complications, Jacheć et al^13^ proposed the categorization of patients undergoing TLE into low risk (0.16%–0.47% risk of major complications), intermediate risk (0.48%–2.46%), high risk (2.52%–11.82%), and very high risk (>11.82%).

This risk stratification model was prospectively applied to a validation cohort of 551 patients. A score of 9.93 was used as a cutoff to indicate a patient with high procedural risk of major complication. The generalizability of this study is limited by the absence of powered sheath use in any of the TLE procedures included.

### EROS (2021)^14^

EROS was built on the risk stratification model devised by Sidhu et al^14^ with modifications on lead dwell times, a more inclusive criterion for chronic kidney disease, and more specific criteria for infectious indication. Patients undergoing TLE were categorized into 3 categories: EROS 1, EROS 2, and EROS 3 (Table 3).

**Table 3:** EROS risk stratification criteria

EROS 1	EROS 2	EROS 3
Pacemaker lead ≤15 y from implant or ICD lead ≤10 y from implant	Pacemaker lead ≤15 y or ICD lead ≤10 y from implant and either: 1. Congenital heart disease 2. Initial implant when the patient was <15 y old 3. Chronic kidney disease and serum creatinine >2 mg/dL 4. Infectious indication for extraction and any 1 of the following: a. White cell count >12 × 10^9^/L b. Positive blood culture c. Vegetation on transesophageal echocardiogram 5. Chronic heart failure and NYHA class IV	Pacemaker lead >15 y from implant or ICD lead >10 y from implant

EROS = ELECTRa registry outcome score; ICD = implantable cardioverter-defibrillator; NYHA = New York Heart Association.

This risk score was retrospectively applied to the ELECTRa registry population, consisting of 3510 patients.^2^ Several findings were made:

1. Compared with EROS 1 and EROS 2, the EROS 3 group was more likely to
  a. Experience major procedure-related complications including death (5.1% vs 1.3%; *P* < .0001)
  b. Experience postprocedural death (0.8% vs 0.2%; *P* = .0449)
  c. Experience all-cause in-hospital major complications including death (OR 2.339; 95% CI 1.439–3.803; *P* = .0006)
  d. Require powered sheaths, including the use of laser (44.9% vs 29.2%; *P* < .0001)
  e. Require a femoral approach (8.3% vs 3.8%; *P* = .0001)
  f. Have a longer procedure time (114 [76–160] vs 80 [55–120] minutes; *P* < .0001)
  g. Be woman
  h. Have a lower body mass index
  i. Fail to achieve clinical success (89% vs 97.7%; *P* < .0001)
2. Compared with EROS1, EROS 2 patients were
  a. More likely to use powered sheaths
  b. More likely to require a femoral approach
  c. Associated with a higher risk of all-cause deaths(2.9 vs 0.5%; *P* < .0001), driven by heart failure and sepsis.
  d. Less likely to be in “high-risk” environment (eg, hybrid laboratory or operating room)

Overall, findings from this study suggest that patients in the high-risk group (EROS 3) would benefit from the presence of immediate surgical backup and should be performed in a hybrid laboratory or operating room owing to the more than 4-fold risk of major complications including death than EROS 2 and 1 combined. The EROS 2 group was more likely to have their TLE performed in a low-risk setting than the EROS 1 group despite a higher risk of requiring powered sheaths and a femoral approach and a higher risk of all-cause in-hospital deaths, suggesting that risk stratification scores may be able to further optimize the planning of TLE in this group. The low-risk group (EROS 1) was less likely to require powered sheaths or a femoral approach compared with EROS 2 and may be performed in the EP laboratory with surgical backup. However, female sex, which has consistently been shown to independently predict major complications, does not form part of the EROS risk score and should be taken into consideration when risk stratifying prospective TLE candidates.^1^^,^^2^^,^^15^, ^16^, ^17^

### Mehta et al (2022)^18^

Mehta et al^18^ investigated the use of machine learning to predict major complications and procedural death associated with TLE. The model was trained on preprocedural factors from the 3155 patients in the ELECTRa registry and subsequently validated on 1151 patients who underwent TLE at St. Thomas’ Hospital, United Kingdom. Multiple machine-learning algorithms were trained and tested, and the best-performing model was compared with the EROS score. Overall, there was a modest improvement in the predictive capability of the machine-learning model for major complications, with the main difference being in improved accuracy in identifying lower-risk patients.

This model by Mehta et al^19^ was vastly improved by the incorporation of data obtained from preprocedural chest radiographs. Imaging biomarkers identified as risk factors by machine learning included the presence of >50% of a defibrillator coil in the SVC, acute angulation of leads in the heart and SVC, and ≥2 overlapping leads in the SVC. Sensitivity (68%–83%), specificity (72%–91%), and area under the curve (0.767–0.962) all improved with the integration of imaging biomarkers into the model.

### Canadian lead extraction risk study (2022)^17^

Bashir et al^17^ retrospectively analyzed 2325 patients who had undergone TLE—predominantly using laser—across 8 Canadian institutions between 1996 and 2016. The authors examined the incidence of cardiac or vascular perforation and 30-day mortality, developing a regression-based model to predict perforation risk. Independent predictors of cardiac or vascular perforation included female sex (OR 3.27; 95% CI 1.91–5.60), diabetes (OR 2.12; 95% CI 1.16–3.86), no previous cardiac surgery (OR 3.33; 95% CI 1.54–7.19), left ventricular ejection fraction of ≥40% (OR 2.81; 95% CI 1.28–6.14), ≥2 leads extracted (OR 2.49; 95% CI 1.23–5.04), and oldest lead age of >8 years (OR 2.64; 95% CI 1.52–4.60). The predictors of 30-day mortality were age (OR 1.04, CI 1.01–1.07), anemia (OR 3.14, CI 1.38–6.61), and infectious indication (OR 3.85, CI 1.38–10.73). Notably, laser extraction was used in 95% of patients. However, this model was not externally validated, and no formal risk classification system was proposed.

Table 4 summarizes and compares the procedural risk models described.

**Table 4:** Comparison between different risk stratification models

Study	Fu et al	Kancharla et al^11^	Afzal^12^ et al “RISE”	Jacheć^13^ et al “SAFeTY” score	Sidhu et al “EROS” score	Mehta et al^18^	Bashir et al^17^ “CLEAR”
Year published	2015	2019	2019	2020	2021	2022	2022
Sample size (n)	652	187	1200	2600	3510	4726	2325
Derivation cohort	Yes	No	Yes	Yes	No	Yes	Yes
Validation cohort	No	Yes	Yes	Yes	Yes	Yes	No
Complete procedural success rate	97%	92.5%	Not reported	95%	95.7%	95.7%	94.5%
Clinical success rate	Not reported	97.9%	Not reported	97.9%	96.7%	96.7%	Not reported
Score components	Low risk Oldest implanted lead ≤1 y old Moderate risk Old lead 1–10 y old or old ICD lead ≤5 y old High risk Oldest PPM lead >10 y old, older ICD lead >5 y old	Intermediate risk PPM lead <10 y from implant or ICD lead <5 y from implant High risk PPM lead >10 y from implant or ICD lead >5 y from implant OR - PPM lead <10 y from implant or ICD lead <5 y from implant + congenital heart disease, initial implant when patient was aged <15 y, hemodialysis, CXR or CT showing calcified SVC or myocardium adjacent to lead, active sepsis or NYHA IV heart failure	Low risk PPM and single-coil ICD <5 y old or dual-coil ICD <3 y old High-risk PPM and single-coil ICD >5 y old, dual-coil ICD >3 y old, or StarFix lead of any age	Composite of: - Sum of dwell times for leads planned for extraction >16.5 y - First implantation under the age of 30 y - Hemoglobin concentration <11.5 g/dL - Female gender - Number of previous CIED procedures	Low risk (EROS 1) PPM lead ≤15 y from implant or ICD lead ≤10 y from implant. Intermediate risk (EROS 2) PPM lead ≤15 y from implant or ICD lead ≤10 y from implant plus any of the following: - Congenital heart disease - Initial implant when the patient was <15 y old - Chronic kidney disease and serum creatinine >2 mg/dL - Infectious indication for extraction - Chronic heart failure and NYHA class IV High risk (EROS 3) PPM lead >15 y from implant or ICD lead >10 y from implant.	Composite of: - LVEF - Lead dwell time - Presence of ICD lead to be removed - Sepsis - Sex - Local infection - Heart failure - Respiratory comorbidity - eGFR	Composite of: - Female sex - Diabetes - LVEF ≥40% - No previous cardiac surgery - ≥2 leads extracted - Oldest lead ≥8 y

CIED = cardiac implantable electronic device; CLEAR = Canadian lead extraction risk; CT = computed tomography; CXR = chest radiograph; ICD = implantable cardioverter-defibrillator; EROS = ELECTRa registry outcome score; LVEF = left ventricular ejection fraction; NYHA = New York Heart Association; PPM = permanent pacemaker; RISE = risk stratification before lead extraction; SVC = superior vena cava.

## Risk prediction for procedural difficulty and need for advanced tools

In addition to risk stratification models for procedural risk, scores to predict the difficulty of TLE and the need for advanced tools have been proposed. Risk stratification models that predict the need for advanced tools may facilitate decisions concerning the most appropriate lead management (extraction or abandonment) and determine the ideal setting for TLE (device laboratory, hybrid laboratory, or operating room).

### Mazzone et al (2013)^20^

Mazzone et al^20^ retrospectively analyzed 210 TLE procedures at a single center and identified the independent predictors of powered sheath use. Using receiver-operator characteristic analysis, cutoff values that best predicted the use of powered sheath were determined. The final prediction score consisted of age of <70.7 years, extraction of ≥2 leads, implant duration of >37 months, and extraction of a high-voltage lead, which was largely in keeping with other studies.^7^^,^^21^, ^22^, ^23^, ^24^ The presence of 0, 1, 2, 3, and 4 risk factors corresponded to 0%, 8.3%, 66.7%, and 86.7% risk of powered sheath use, respectively.

### Lead extraction difficulty score (2014)^25^

Bontempi et al^25^ prospectively analyzed 469 patients undergoing TLE at a single center to identify the risk factors for procedural difficulty, defined as the whole procedure fluoroscopy time exceeding the 90th percentile. The independent predictors were the number of extracted leads, combined age of leads to be extracted, presence of dual-coil ICD leads, and absence of vegetation. These predictors were used to form the lead extraction difficulty score, where:

LEDScore=numberofleadstoextract+ageofoldestleadtoextract+1(ifdualcoilICDleadtobeextracted)−1(ifvegetationconfirmedalongtheleadbody).

A cutoff score of 10 was identified as the score with the highest combined sensitivity and specificity (78.3% and 76.3% respectively) for procedural difficulty using receiver-operator characteristic analysis.

### MB score (2020)^21^

Building on the 2 previous risk stratification scores, a total of 973 TLE procedures across 2 centers were retrospectively randomized to the derivation cohort (n = 487) and validation cohort (n = 486).^21^ The derivation cohort was analyzed for predictors of advanced procedure, defined as the use of powered sheaths or femoral snaring. The independent predictors identified from the derivation cohort were then used to form the MB score, which comprised lead age, number of leads to be extracted, and type of lead (passive fixation or ICD lead). This score was tested against the validation cohort and was identified as an independent predictor of advanced extraction (OR 2.40; 95% CI 2.02–2.86; P < .001) and demonstrated an area under the curve of 0.80. A score of 6 was associated with a 96.4% chance of advanced extraction.

### Multicenter imaging in lead extraction study (2019)

The multicenter imaging in lead extraction study (MILES) was a prospective, blinded, multicenter study by Patel et al,^26^ which aimed to evaluate whether the degree of fibrosis from preprocedural cardiac CT scan in patients undergoing TLE would predict the need for the use of powered sheaths in the SVC. Degrees of fibrosis were graded between 1 (least fibrosis) and 4 (most fibrosis). This was built on previous, smaller retrospective studies evaluating the association of different imaging characteristics with difficult extraction procedures.^27^ In total, 200 patients were included in the MILES. A significantly higher proportion of patients with a greater degree of fibrosis in the SVC (scores 3 and 4) required powered sheath than patients with a lower degree of fibrosis (scores 1 and 2) (67.8% vs 38.5%, respectively; *P* < .01).

## Postprocedural risk stratification

Although procedural mortality has declined over the last few decades with advancement in lead extraction technology and operator experience, postprocedural mortality across all indications remains substantial, ranging from 1.6% to 5.6% at 30 days and from 4.5% to 26% at 1 year after TLE, despite high procedural and clinical success rates.^4^, ^5^, ^6^, ^7^^,^^17^^,^^28^^,^^29^ This underscores the need to examine the drivers of postprocedural mortality.

## 30-day mortality

### Brunner et al (2015)^30^

**Figure 2:**
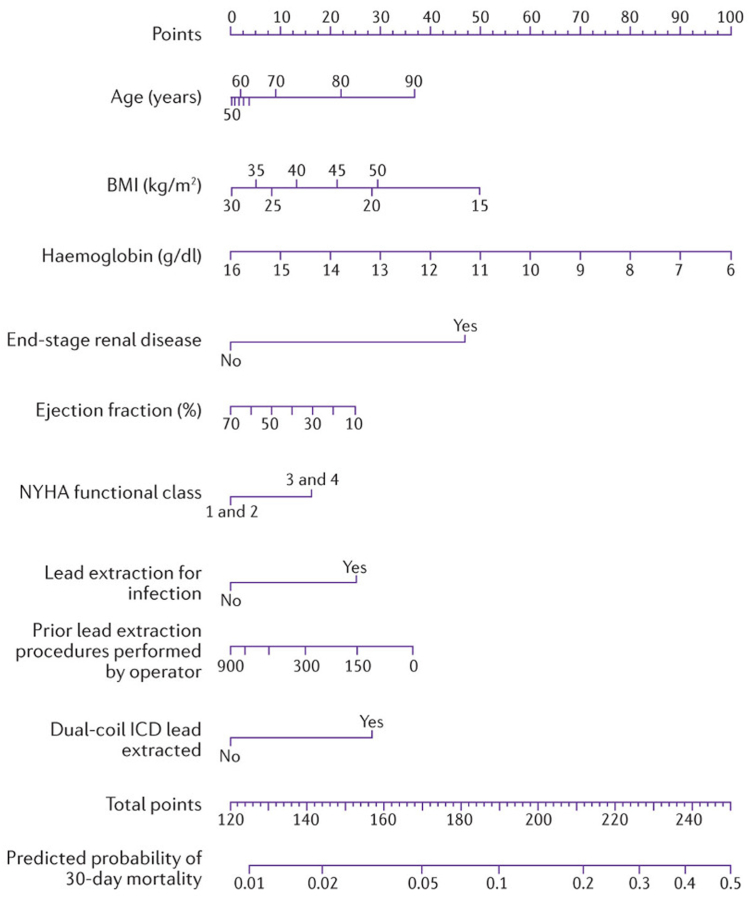
Nomogram for prediction of 30-day all-cause mortality. Adapted from Elsevier, source: Brunner et al.^30^ BMI = body mass index; ICD = implantable cardioverter-defibrillator; NYHA = New York Heart Association.

Brunner et al^30^ proposed a preoperative risk score to predict the risk of 30-day all-cause mortality after TLE. A retrospective analysis of 2999 patients undergoing TLE was performed to identify the risk factors for 30-day all-cause mortality, and a nomogram was subsequently generated with risk factors including age, body mass index, hemoglobin, presence of end-stage renal disease, ejection fraction, New York Heart Association class, infectious indication, operator experience, and presence of dual-coil ICD to be extracted (Figure 2). The 30-day all-cause risk can be calculated using a simplified online calculator with a concordance index of 0.86. The overall mortality at 30 days was 2.3%, and most deaths (89%) were not procedure related. This reinforced the notion that postprocedural deaths may be affected by separate drivers from procedural deaths.

## One-year mortality

### IKAR risk score (2017)

Oszczygiel et al^31^ conducted a retrospective analysis of 130 patients who underwent TLE. A multivariate analysis was performed to identify risk factors for 1-year mortality, which found them to be infectious indications for TLE, age, chronic kidney disease, and presence of high-voltage leads to be removed. These were used to form the IKAR scoring system based on risk factors including infectious indication (score 1), kidney dysfunction (score 2), age of ≥56 years (score 1), and high-voltage lead to be removed (score 1). Those with IKAR scores 3–5 had almost 4-fold higher risk of 1-year mortality than those with IKAR scores 0–2.

## Improving outcomes with risk stratification models

To date, no single risk stratification model encompasses all predictors of major complications and mortality associated with TLE. The recurring predictors of periprocedural major complications and mortality are infectious indication, female sex, chronic kidney disease, implant duration of >10 years, number of leads, and symptomatic heart failure.^1^^,^^2^^,^^15^, ^16^, ^17^ The machine-learning model by Mehta et al,^18^ trained on the ELECTRa registry (n = 3155) and validated in an independent cohort of 1151 patients, incorporates both lead and nonlead parameters and preprocedural imaging data, making it the most comprehensive model to date. However, this model is not yet publicly available. In its absence, the EROS model—also validated using the ELECTRa registry—serves as a robust alternative. The EROS model integrates traditional lead-related risk parameters while also accounting for nonlead-related factors such as infectious indication, renal impairment, chronic heart failure, congenital heart disease, and age at implantation. Beyond predicting major complications, the EROS score also stratifies risk for powered sheath use and a femoral approach. Its limitation lies in the exclusion of female sex, which has been consistently found to be a predictor of major complications and mortality associated with TLE.^1^^,^^32^ Therefore, we recommend the use of the EROS model in conjunction with female sex to optimize TLE risk stratification.

## Location of TLE and availability of surgical backup

Determining the appropriate setting to perform TLE is crucial. An operating room affords immediate access to surgical equipment in the event of a major complication necessitating cardiac surgery and a surgically trained team familiar with operative tools and workflow. This is important because a surgical response time of more than 10 minutes in the event of an SVC tear has been associated with a high risk of mortality.^33^ In contrast, an EP laboratory offers superior fluoroscopic imaging and specialized TLE equipment and reduces demand on surgical personnel and operating room resources. A hybrid laboratory integrates the benefits of both settings but remains limited by the need for surgical staffing and scheduling constraints owing to its high demand across specialties. Scheduling issues owing to limited operating room or hybrid laboratory availability can lead to delays in TLE, which in turn leads to greater mortality.^34^ In a study by Pokorney et al,^35^ the 1-year mortality rate for those who did not have their devices extracted within 30 days was 32.5%.

Although current risk stratification models recommend hybrid laboratories or operating rooms for high-risk TLEs, our subanalysis of the ELECTRa registry showed no significant difference in major complications, procedural mortality, vascular tears, or cardiac avulsions between procedures performed in EP laboratories and those in hybrid laboratories. This was despite EP laboratory patients being older and having lower left ventricular ejection fraction, higher New York Heart Association class, more comorbidities, and greater use of powered sheaths. These findings support the safety of performing TLEs in the EP laboratory, even in high-risk patients. Consistently, the German laser extraction registry—where all procedures were conducted in hybrid laboratories with full surgical backup—reported similar complication and mortality rates to ELECTRa, in which more than a third of TLEs occurred in the EP laboratory.

**Figure 3:**
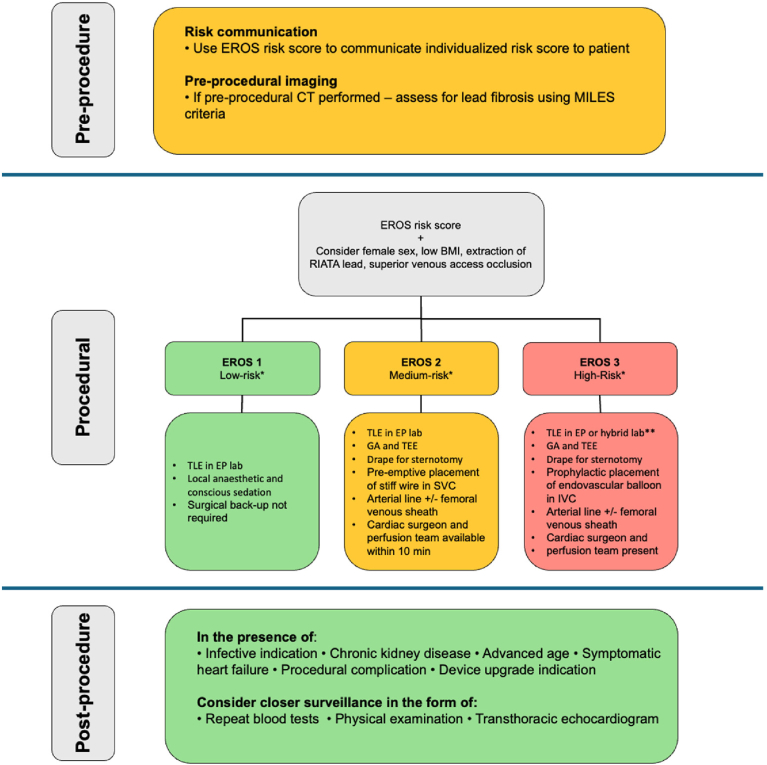
Flowchart illustrating the suggested TLE algorithm guided by risk models and risk factors. ∗Overall risk should take into account EROS in conjunction with female sex. CT = computed tomography; EP lab = electrophysiology laboratory; EROS = ELECTRa registry outcome score; GA = general anesthetic; SVC = superior vena cava; TEE = transesophageal echocardiogram; TLE = transvenous lead extraction.

Using the EROS risk stratification model, patients classified as low (EROS 1) or intermediate risk (EROS 2) of major complications can safely undergo extraction procedures in the EP laboratory.^14^ Procedures classed as EROS 2 should be performed with surgical backup present in the building and able to respond within 10 minutes of a major complication. Procedures classified as EROS 3 may benefit from being performed either in a hybrid laboratory or an EP laboratory with adequate provisions for the perfusion team and surgical equipment and a surgeon being physically present in the room. A suggested algorithm for determining the appropriate level of care for different risk groups is presented in Figure 3.

## Use of an endovascular occlusion balloon

SVC tear is often the most lethal complication during TLE, with a mortality rate exceeding 50% without any bridging therapy to emergent surgery.^36^ The use of an endovascular balloon as a bridging measure to surgical repair has been shown to significantly reduce mortality.^37^ However, the use of an endovascular balloon adds to the already substantial cost of a TLE.^38^ Therefore, judicious use of the endovascular occlusion balloon only in patients at high risk of SVC injury is paramount. For patients classified as EROS 2, we recommend the preemptive placement of a stiff wire in the inferior vena cava to allow rapid balloon deployment in the event of an SVC tear. In the EROS 3 group, we recommend the prophylactic placement of the endovascular balloon in the SVC. This approach is supported by the findings of Tsang et al^39^ who found that the time to deployment was 14 seconds for a prophylactically placed balloon vs 120 seconds when opened from a package, equating to approximately 700 mL blood loss in the setting of SVC tear.^40^

## Risk communication

Despite the low overall complication and mortality rates reported in recent TLE registries, it remains essential to communicate individualized procedural risk to patients, given that outcomes can vary widely. For instance, patients classified as EROS 3 have a 5.1% risk of major procedural complications, including death, compared with 1.3% in the EROS 1 and 2 groups. Rather than relying on general procedural statistics, we recommend using the EROS model to convey patient-specific risk. This approach facilitates accurate risk communication, supports shared decision making, and helps patients form realistic expectations regarding prognosis and procedural success.

## Preprocedural imaging

In patients undergoing preprocedural CT imaging, the assessment of lead fibrosis—based on criteria from the MILES—may provide additional risk stratification beyond the EROS model in facilitating the prediction of powered sheath use and further optimizing procedural planning and preparation.^27^

## Postprocedural care and monitoring

There are currently no formal guidelines defining the nature or duration of follow-up after TLE. The 2017 HRS expert consensus on lead extraction mentions the use of chest radiograph to rule out hemothorax and pneumothorax and transthoracic echocardiogram to screen for tricuspid valve injury, lead remnants, and pericardial effusion within 24 hours of extraction but does not proffer this as a formal recommendation.^9^ The 2018 EHRA expert consensus statement on lead extraction recommends a minimum follow-up period of 30 days but does not specify the nature of follow-up.^8^ Both HRS and EHRA acknowledge the significant postprocedural mortality despite high procedural and clinical success rates.

**Figure 4:**
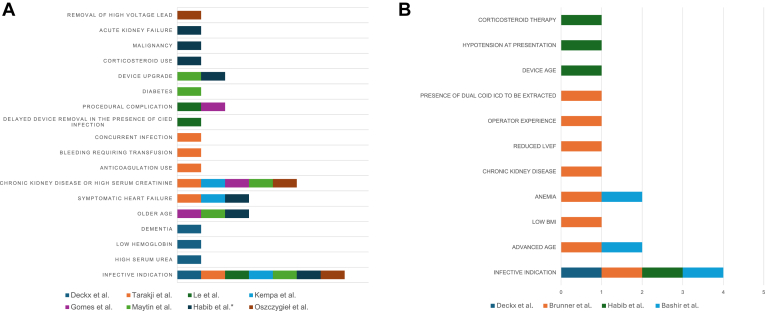
Pooled independent predictors of (A) 1-year mortality and (B) 30-day mortality after TLE. Bar colors denote different studies. ∗Duration of long-term mortality was not defined in this study. BMI = body mass index; LVEF = left ventricular ejection fraction; TLE = transvenous lead extraction.

Robust postprocedural risk stratification models remain limited. The IKAR scoring model, developed from a small cohort of 130 patients, requires validation in a larger cohort before it can be reliably applied. By far the most common predictor of postprocedural mortality is infectious indication, followed by chronic kidney disease, advanced age, symptomatic heart failure, and anemia.^3^, ^4^, ^5^, ^6^, ^7^^,^^17^^,^^28^^,^^29^ The pooled predictors of 30-day and 1-year mortality after TLE are presented in Figure 4. Patients with 1 or more of these risk factors may benefit from closer surveillance in the form of repeat blood tests, physical examination, and, when clinically indicated, follow-up transthoracic echocardiogram to detect late complications such as pericardial effusion, tricuspid regurgitation, or missed retained lead fragments. Therefore, a minimum follow-up period of 1 year should be considered after all TLE procedures.

## Conclusion

The risk of major procedural complications and mortality during TLE is highly variable and depends on individual patient and procedural factors. Risk stratification models allow for personalized risk assessment, improving resource allocation, guiding the use of specialized tools such as powered sheaths and vascular occlusion balloons, reducing surgical response times, and supporting informed patient discussions. They also help determine the appropriate intensity and duration of postprocedural follow-up. Although acute procedural success rates for TLE are high, postprocedural mortality remains a significant concern. Currently, no validated model exists to accurately predict postprocedural outcomes. Common risk factors associated with increased mortality include infectious indication, symptomatic heart failure, advanced age, and chronic kidney disease. Patients presenting with these factors are likely to benefit from closer postprocedural monitoring.

## Disclosures

N.W., F.d.V., A.C.L., and S.H. are supported by the Wellcome/Engineering and Physical Sciences Research Council Centre for Medical Engineering (WT203148/Z/16/Z). N.W. has received funding from the British Heart Foundation (FS/CRTF/22/24362) and travel funding from EBR Systems. S.N. acknowledges support from the UK Engineering and Physical Sciences Research Council (EP/M012492/1, NS/A000049/1, EP/P01268X/1), the British Heart Foundation (PG/15/91/31812, PG/13/37/30280, SP/18/6/33805), US National Institutes of Health (R01-HL152256), and European Research Council (ERC PREDICT-HF 864055). C.A.R. receives research funding and/or consultation fees from Abbott, Medtronic, Boston Scientific, Spectranetics, EBR Systems, and MicroPort.
